# Detection of Antibodies Against Canine Circovirus in Naturally and Experimentally Infected Canines by Recombinant Capsid Enzyme-Linked Immunosorbent Assay

**DOI:** 10.3389/fvets.2020.00294

**Published:** 2020-05-28

**Authors:** Zheng Wang, Yunjia Shi, Yu Wang, Lili Zhao, Xingyang Cui, Shanshan Wen, Hanghang Liu, Wen Cui, Hongyan Chen, Junwei Ge

**Affiliations:** ^1^College of Veterinary Medicine, Northeast Agricultural University, Harbin, China; ^2^State Key Laboratory of Veterinary Biotechnology, Heilongjiang Provincial Key Laboratory of Laboratory Animal and Comparative Medicine, Harbin Veterinary Research Institute, Chinese Academy of Agricultural Sciences, Harbin, China; ^3^Northeastern Science Inspection Station, China Ministry of Agriculture Key Laboratory of Animal Pathogen Biology, Harbin, China

**Keywords:** canine circovirus, serological detection, capsid protein, indirect ELISA, pathogenicity

## Abstract

Canine circovirus (CanineCV), a new pathogen, was found to be associated with canine hemorrhagic diarrhea, vasculitis, granulomatous lymphadenitis, and acute gastroenteritis. Although CanineCV was highly positive rate in diarrhea cases, its pathogenicity remains controversial. In this study, the seroprevalence and associated risk factors of CanineCV infection among domestic dogs in northeastern China was investigated by an indirect enzyme-linked immunosorbent assay (iELISA) based on recombinant capsid protein. Results revealed the proposed iELISA had no cross-reactivity with other related pathogens, and yielded good diagnostic values. Then, to evaluate the rCap iELISA, this study applied it to detect antibodies against CanineCV in 1,047 clinical serum samples obtained from northeastern China in 2016–2017. Results showed the positive rates in the five cities of Jilin, Liaoning, and Heilongjiang provinces ranged from 22.22 to 42.29%. Statistical analysis shows a significant difference in age between dogs <3 months old with respect to the >1-year-old dogs (*p* = 0.005), that is, the CanineCV infection was more frequently identified from older dogs. In the artificially infected experiment, the dogs developed seroconversion after 9 or 12 days and the main way of virus excretion was through feces. More interestingly, among the 32 ELISA-positive serum samples, 34.75% samples tested positive for the CanineCV DNA by qPCR, far higher than that in ELISA-negative serum samples (5.26%, 2/38). This report is the first to demonstrate that CanineCV infection is common in the dog population in northeastern China. The results showed obvious differences in the positive rate associated with diarrhea, age, but not with different cities. This study also provide basis for evaluating the pathogenic potential of CanineCV. But, the pathogenicity, the relationship between antibody level and immune protection, and the harmful effects of this virus remain to be established.

## Introduction

Circoviruses are small, non-enveloped icosahedral viruses that have a diameter of 16–26 nm and a single-stranded circular DNA genome with a length of 1.7–2.1 kb and a major structural protein (1). These viruses belong to the genus *Circovirus* in the family Circoviridae. Circoviruses have been identified in numerous species associated with diverse clinical disorders, including weight loss, respiratory distress, diarrhea, and lethal diseases. These viruses are also associated with lymphoid depletion and immunosuppression in type 2 porcine circoviruses, beak and feather disease virus, and pigeon circovirus (2–4). In 2012, a canine circovirus (CanineCV) was first detected in serum samples from several dogs with no clinical history (5). CanineCV was then detected to be associated with canine vasculitis and/or hemorrhagic diarrhea and granulomatous lymphadenitis (4, 6). A retrospective study revealed that this virus has been circulating in dogs since 2009 (6). Molecular epidemiological analyses showed the genetic recombination of CanineCV genomes (7, 8).

The DNA genome of CanineCV with length of 2 kb contains two major open reading frames (ORFs) (5) as follows: ORF1/V1, or the rep gene, which encodes a replicase-associated protein required for viral replication (303 amino acids [aa]); and ORF2/C1, the cap gene, which encodes viral capsid proteins that participate in the host immune responses (270 aa). Although the detection results showed the high positive rate of the virus in the fecal samples from diarrheal dogs (9, 10), the pathogenicity remains controversial. Further studies are required to better understand the disease burden, serological surveys, risks, and dynamics of this infection.

Although conventional polymerase chain reaction (PCR), electron microscopy, immunohistochemistry, *in situ* hybridization, and quantitative real-time PCR (qPCR) (6, 9, 11) have been used to identify CanineCV infections, no specific serological method has been developed for diagnosis.

This study aims to develop a sensitive, specific, and convenient method for rapid detection of virus-specific antibodies in serum and determination of the seroprevalence and associated risk factors of CanineCV infection among domestic dogs in northeastern China. An indirect enzyme-linked immunosorbent assay (iELISA) was developed using the Capsid protein (cap) expressed in *Escherichia coli* as a coating antigen. The proposed iELISA had no cross-reactivity with other related pathogens. This assay was validated by comparing the obtained results with those of Western blot analysis. The applications of iELISA for clinical detection of experimentally infected minks were also described.

## Materials and Methods

### Ethics Approval

This study was carried out in accordance with the recommendations of the Guide for the Care and Use of Laboratory Animals of the Ministry of Health, China; and was approved by Harbin Veterinary Research Institute (approval number Heilongjiang-SYXK-2006-032). Samples for laboratory analysis were collected from animals, while avoiding unnecessary pain and suffering. The owners provided written consent for sample collection, and the sampling locations were not privately owned or protected in any way. The studies did not involve endangered or protected species.

### Blood Samples

Eight healthy dogs from a laboratory animal center and three sera from fetus dog obtained by cesarean section were sampled to represent negative-control dog sera. The laboratory animal center was selected for CanineCV-free on the basis of prior negative testing with PCR once a year for the last 3 years.

A total of 759 serum samples were collected from healthy, diseased, or dead dogs from more than 30 animal clinics in Harbin City, Daqing City, and Mudanjiang City, Heilongjiang Province, in Changchun City, Jilin Province and Shenyang City, Liaoning Province in China from May 2016 to April 2017.

A total of 277 serum samples were collected from the Animal Teaching Hospital of the College of Veterinary Medicine, Northeast Agricultural University. Complete records including the information of owners, age, breed, disease history, clinic manifestations, and treatment were obtained.

The specificity of the iELISA was examined using the positive canine serum samples of canine distemper virus (CDV), canine parvovirus (CPV2), canine adenovirus type 2 (CAV2), and rabies virus (RV), which were obtained from Dr. Jiang Qian and Dr. Jinying Ge (12). All serum samples were stored at −20°C until use.

### Expression, Purification, and Identification of the rCap Protein

The truncated *cap* gene (121–813 nt, deleted 120 nt at the 5′ terminal) was expressed as a His-tagged protein in *E. coli* with the expression vector pET-32a (Novagen). Canine circovirus strain XF16 obtained previously by our team (GenBank accession No. MF797786) was used as template. The gene was amplified through PCR with the forward primer 5′-UGGATCCLINECTGACAGCTGATTG−3′ and the reverse primer 5′-UCTCGAGLINETTACAACTGGCG−3′. The PCR product was digested with *Bam*HI and *Xho*I, and cloned into pET-32a vector. The resulting recombinant plasmid pET-32a-cap was sequenced by Comate Biotech., Changchun, China and transformed into *E. coli* Rosetta cells. The recombinant *E. coli* Rosetta cells were induced to express his-tagged recombinant protein and purified using protein purification kit (Qiagen, Hilden, German) according to the manufacturer's instructions. rCap was identified through Western blot by utilizing HRP-conjugated mouse anti-His MAb (Sigma, Missouri, USA) as antibodies.

To evaluate the potential cross-reactivity of the rCap protein in Western blot, we used the positive canine serum samples of CAV2, CPV2, RV, and CDV. Sera from the laboratory animal center and those from fetus dog obtained by cesarean section were used as negative controls. These samples were assayed in duplicate.

### Western Blot Analysis for Screening Positive and Negative Serum Samples

Western blot analysis was performed following the established procedures. After electrophoresis, the purified rCap proteins and pET-32a vector proteins as controls were electrotransferred onto nitrocellulose blotting membranes (Pall Corporation, USA). After blocking the membranes by using 5% skimmed milk-PBS and washing three times with PBS-T (0.05% Tween 20 dissolved in PBS), the membranes were incubated for 2 h at 37°C with serum samples diluted in 1:100. The membranes were washed three times with PBS-T, and further incubated for 1 h with horseradish peroxidase-conjugated rabbit anti-dog IgG (Bioss antibodies, Beijing, China) at a dilution of 1:5,000. After washing three times, the bands were detected using DAB reagents (Biotopped, Beijing, China).

### Development of Indirect ELISA

iELISA was developed based on purified rCap according to the published protocol (13–16). The optimum concentrations of coating antigen, serum dilution, and horseradish peroxidase-conjugated rabbit anti-dog IgG were determined through checkerboard serial-dilution analysis.

In brief, rCap was serially diluted two-fold from 1 to 0.03125 μg/ml in 0.05 M Na bicarbonate/carbonate buffer. The CanineCV-positive and CanineCV-negative sera confirmed by western blot were also serially diluted two-fold from 1:25–1:400 and used to optimize rCap ELISA. The optimal antigen concentration and serum dilution were determined to be the conditions that resulted in the greatest ratio of OD_450_ nm values between positive and negative serum (P/N value) with the positive control serum yielding an absorbance near 1.5. After the optimal antigen concentration and serum dilutions were established, checkerboard titrations were performed to screen the optimal working dilutions of the conjugate rabbit anti-dog IgG, blocking buffer, and reaction time. The conjugate rabbit anti-dog IgG was added to the plate at ratios of 1:1,000, 1:2,000, 1:5,000, and 1:10,000 to determine the optimal dilution of conjugated antibody. The conditions that yielded the highest OD_450_ ratio between P/N value were considered optimal. The cut-off value was calculated from the mean OD of the negative samples in Western blot (*n* = 60) plus 3 standard deviation (SD).

Intra-assay (within-plate) reproducibility and inter-assay (between-run) reproducibility were assessed as previously described by our group (16). Mean OD, SD, and coefficient of variation (CV) were calculated.

The correlation between rCap ELISA and Western blot was examined using 143 canine serum samples.

### Application of iELISA for an Epidemiological Survey on CanineCV Infection

To estimate the seroprevalence of CanineCV in the canine population, we randomly collected serum samples (*n* = 759) from more than 30 animal clinics in five cities in northeastern China from 2016 to 2017. Based on the iELISA procedure, the antibody titers were determined through double dilution method.

### Real-Time PCR for the Detection of CanineCV in the Serum Samples

To quantify CanineCV DNA loads, 70 serum samples, including 32 positive samples and 38 negative samples, were selected for real-time PCR analysis (9). Two microliter of sample DNA was used in the qPCR. All samples and controls were tested per triplicate.

### Experimental Infection

Because we could not get the sample that only had CanineCV infection, we had to use a CPV2 and CanineCV mixed infection sample to operate experimental infection. The liver from this case was homogenized, frozen and thawed three times, centrifuged at high speed, and then took the supernatant for later use.

The experimental dogs were screened for CPV-1, CAV, and CDV by (RT-)PCR and found to be negative, and antibody to CPV2 lower 2^2^ (HI titer), negative to CanineCV. Five 30-day-old sibling puppies were randomly divided into two groups. Three animals in virus-inoculated group were infected by intragastric (i.g.) inoculation with the virus (contain 10^8^ copies of CanineCV DNA tested by quantitative real-time PCR) diluted in PBS. Simultaneously, 2 puppies were inoculated with PBS (I.g.) as a control. For 15 days, all puppies were monitored daily for clinical signs of disease. Fecal and nasal swabs were collected from all animals to assess viral shedding at days 0, 2, 4, 6, 8, 10, 12, 14, and 15 post-infection (dpi). Sera were collected from dogs every 3 days to determine the antibody.

## Results

### Expression and Identification of the rCap Protein

**Figure 1:**
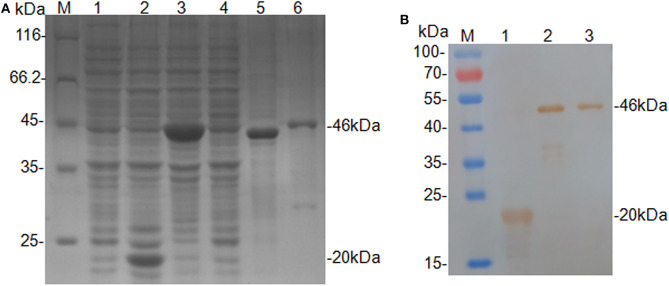
Expression and identification of the rCap fusion protein. **(A)** SDS-PAGE analysis: Lane M, protein molecular weight marker; Lane 1, Rosetta control; Lane 2, pET-32a vector control; Lane 3, pET-32a-cap bacterial lysate; Lane 4, supernatant from pET-32a-cap bacterial lysate; Lane 5, the pellet of pET-32a-cap bacterial lysate; Lane 6, purified fusion protein. **(B)** Western blot analysis: Lane M, protein molecular weight marker; Lane 1, pET-32a vector control; Lane 2, pET-32a-cap bacterial lysate; Lane 3, purified fusion protein.

The truncated CanineCV *cap* gene was successfully amplified from the genomic DNA of XF16 strain, and then cloned into the expression vector pET-32a. The insert yielded 100 and 92.93% nucleotide identities with CanineCV XF16 (GenBank accession No. MF797786) and UCD3-478 (GenBank accession No. KC241983.1), respectively. The recombinant protein (rCap) was expressed successfully in an insoluble form and His-tagged fusion protein corresponding to the predicted size (46 kDa). The fusion protein was purified using a Ni-NTA His bind resin column identified through SDS-PAGE analysis (Figure 1A), and confirmed by Western blot analysis by using anti-His-tag monoclonal antibody (Figure 1B). The 46 kDa rCap protein reacted strongly with the anti-His-tag monoclonal antibody in the Western blot.

**Figure 2:**
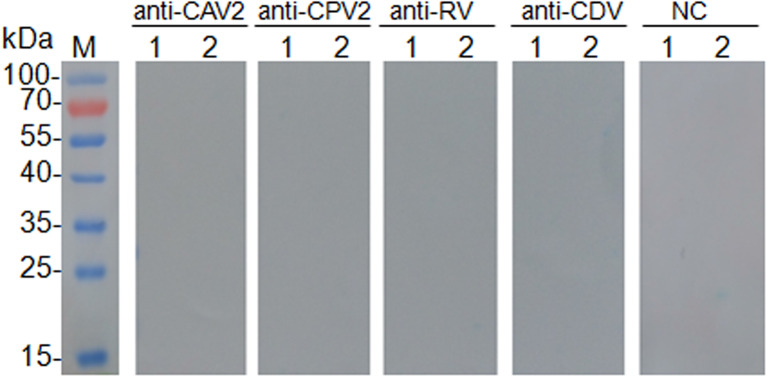
Results of cross test of Western blot was performed with canine adenovirus type 2 positive sera (anti-CAV2), canine parvovirus positive sera (anti-CPV2), rabies virus positive sera (anti-RV), canine distemper virus positive sera (anti-CDV), and CanineCV negative sera control (NC). Lane M, protein molecular weight marker; Lane 1, pET-32a vector control; Lane 2, purified protein rCap.

No cross-reactions were detected in Western blot analysis involving negative sera and antisera against related pathogens, such as CAV2, CPV2, RV, and CDV (Figure 2). This result confirmed the antigenic specificity of rCap protein, while could be used as antigen to detect specific antibodies against CanineCV.

### Development of the rCap ELISA

The optimal coating antigen concentration and serum dilution in the iELISA were 0.0625 μg/ml and 1:100, respectively (Table 1). Other factors were optimized as follows: the optimum blocking buffer for this assay was 5% skimmed milk; the optimal working dilution of the conjugate rabbit anti-dog IgG was 1:5,000, and the optimum incubation periods for the serum samples or the detection reagents were set at 90 or 60 min, respectively.

**Table 1:** Ratio of CanineCV-positive serum to CanineCV-negative serum for the optimization of assay conditions for the ELISA against recombinant rCap antigen.

**Serum dilution**	**Concentration of rCap antigen (μg/well)**					
	**1**	**0.5**	**0.25**	**0.125**	**0.0625**	**0.03125**
1:25(+)	2.748	2.589	2.512	2.465	2.336	1.998
1:25(–)	0.121	0.109	0.112	0.125	0.132	0.141
1:50(+)	2.640	2.558	2.293	2.223	1.964	1.926
1:50(–)	0.110	0.109	0.145	0.165	0.2	0.251
1:100(+)	1.551	1.402	1.384	1.641	**1.596**	2.185
1:100(–)	0.199	0.196	0.152	0.15	**0.122**	0.122
1:200(+)	1.876	1.763	1.753	1.649	1.583	1.674
1:200(–)	0.071	0.076	0.082	0.104	0.118	0.123
1:400(+)	1.696	1.781	1.798	1.891	1.921	1.849
1:400(–)	0.089	0.110	0.111	0.097	0.109	0.093

*Optimum values with the best positive-to-negative ratio are highlighted*.

The mean ± SD OD_450_ value for 60 serum samples tested negative by Western blot in the rCap iELISA was 0.134133 ± 0.043505, resulting in a cutoff value of 0.264 (mean + 3SD). Serum samples with an OD_450_ > 0.264 were judged as positive for CanineCV. No cross-reactions were detected by the rCap iELISA by using antisera against CAV2, CPV2, RV, CDV, *E. coli*, and *S. typhimurium*. The OD_450_ values ranged from 0.118 to 0.019 (Table 2). The developed rCap iELISA possessed high specificity for CanineCV antibody detection.

**Table 2:** Results of cross reaction test of direct ELISA.

**Serum sample**	**Value of OD_**450**_**
DogCV(+)^a^	1.369 ± 0.025
DogCV(–)^a^	0.099 ± 0.012
Blank control	0.074 ± 0.007
Rabies virus	0.100 ± 0.013
Canine parvovirus 2	0.118 ± 0.009
Canine distemper virus	0.098 ± 0.018
Canine adenovirus type 2	0.109 ± 0.014
*Escherichia coli*	0.089 ± 0.037
*Salmonella typhimurium*	0.019 ± 0.01

a *The serum showed positive or negative by Western blot*.

*All samples and controls were repeated 3 times*.

The reproducibility of rCap iELISA was evaluated by comparing CVs of each serum sample in several tests. The intra-assay CV of three positive and three negative serum samples determined through Western blot ranged from 0.56 to 5.44% (Table 3), with a median of 4.03%. The inter-assay CV of these samples varied from 4.15 to 9.32%, with a median of 7.31%. Hence, rCap ELISA was repeatable and yielded low and acceptable variation levels.

**Table 3:** Repeatability and reproducibility analysis of indirect ELISA.

**Sample**	**Intra-assay(OD**_****450****_**)**						**Inter-assay(OD**_****450****_**)**					
	**1**	**2**	**3**	**Mean**	***SD***	**CV%**	**1**	**2**	**3**	**Mean**	***SD***	**CV%**
1	1.613	1.51	1.502	1.541	0.061	4.02	1.261	1.300	1.197	1.252	0.052	4.15
2	1.074	0.996	0.970	1.013	0.054	5.34	1.156	1.002	0.989	1.049	0.093	8.86
3	0.879	0.907	0.815	0.867	0.047	5.44	0.991	0.899	0.881	0.924	0.059	6.39
4	0.073	0.068	0.073	0.071	0.002	4.04	0.078	0.069	0.081	0.076	0.006	8.22
5	0.058	0.06	0.061	0.059	0.001	2.56	0.059	0.058	0.064	0.061	0.003	5.33
6	0.100	0.101	0.100	0.100	0.0005	0.56	0.096	0.101	0.084	0.094	0.008	9.32

### Comparison of iELISA With Western Blot

A total of 143 serum samples were tested by the rCap ELISA in comparison with Western blot. As shown in Table 4, among the 59 Western blot canine positive serum samples, 50 were classified as positive with rCap iELISA; of the 84 Western blot negative serum samples 79 were classified as negative. Fourteen samples presented discordant results between the assays. Therefore, the sensitivity and specificity of the rCap ELISA were 84.75 and 94.05%, and the total coincidence of iELISA and Western blot was 90.21% (Table 4). A κ coefficient of 0.796 (95% CI 0.688–0.884) was calculated, revealing a good agreement between rCap iELISA and Western blot analysis.

**Table 4:** Comparison of the ELISA and Western blot test.

**ELISA results**	**Western blot**		**Total**
	**Positive**	**Negative**	
Positive	50	5	55
Negative	9	79	88
Total	59	84	143
Total (% coincidence)	84.75 (50/59)	94.05 (79/84)	90.21 (129/143)

### Application of iELISA for Epidemiological Surveys on CanineCV Infection

To evaluate the rCap iELISA, we applied it to detect antibodies against CanineCV in 759 sera obtained from more than 30 animal clinics in five cities in Heilongjiang, Jinlin and Liaoning Province in northeastern China in 2016–2017. As shown in Table 5, the positive rate of CanineCV-specific antibodies was 39.82% (417/1,047) in all the sera. These sera were found positive by iELISA with an OD range of 0.266–1.923 (mean 0.321; *S.D*. 0.186). The seroprevalence rates of CanineCV were 42.29% in Harbin City, 38.46% in Daqing City, and 25.53% in Mudanjiang City, Heilongjiang Province, 22.22% in Changchun City, Jilin Province and 42.27% in Shenyang City, Liaoning Province. Statistical analysis shows no significant difference between different cities (*p* = 0.367).

**Table 5:** Summary of the CanineCV results of the sera tested by iELISA.

**No**.	**Sera type**	**Positive/total**	**iELISA**	***P*-value**
			**Positive rate**	
1	Sera from laboratory animal center	0/8	0	–
2	Sera from cesarean section fetus dog	0/3	0	–
3	Location			0.367
	Sera collected from Shengyang City	41/97	42.27%	
	Sera collected from Daqing City	25/65	38.46%	
	Sera collected from Harbin City	225/532	42.29%	
	Sera collected from Changchun City	4/18	22.22%	
	Sera collected from Mudanjiang City	12/47	25.53%	
4	Sera collected from Animal Teaching Hospital			
	Status			0.007
	Sera from dogs without diarrheic disease in the past half year	9/39	23.08%	
	Sera from dogs Suffered diarrheic disease in the past half year	54/118	45.76%	
	Age			0.016
	Sera from young pups <3 months old	11/42	26.19%	
	Sera from >1-year-old dogs	36/78	46.15%	
	Overall rate	417/1,047	39.82%	

To evaluate the correlation between the samples positive for CanineCV and different diarrheal history and age, we also screened antibodies to anti-CanineCV infection in 277 serum samples collected from the Animal Teaching Hospital of the College of Veterinary Medicine, Northeast Agricultural University using iELISA (Table 5). In the 157 serum samples from dogs with or without diarrheic disease in the past half year, 45.76% (54/118) and 23.08% samples (9/39) were positive for CanineCV antibodies. By using chi-square test, the correlation of the diarrhea history with a positive test for CanineCV antibodies was calculated. Among the 42 dogs <3 months old, 26.19% is CanineCV antibody-positive, while 36 (46.15%) in the 78 sera from >1-year-old dogs are positive. Statistical analysis shows a significant difference in age between dogs <3 months old with respect to the >1-year-old dogs (*p* = 0.005), that is, the CanineCV infection was more frequently identified from older dogs.

**Figure 3:**
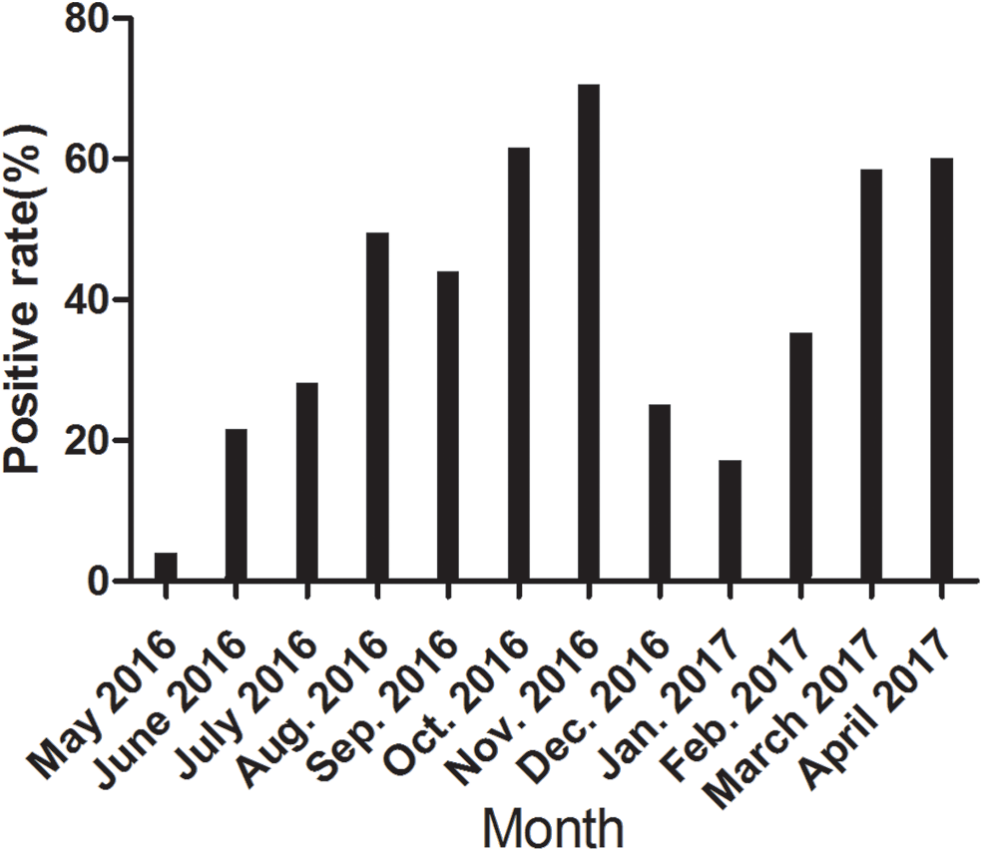
The positive rate of CanineCV antibody every month from May 2016 to April 2017.

We further detected the positive rate of CanineCV antibody every month from May 2016 to April 2017. As shown in Figure 3, the incidence varied greatly monthly, with no unique pattern. The incidence rates varied significantly in different months.

**Figure 4:**
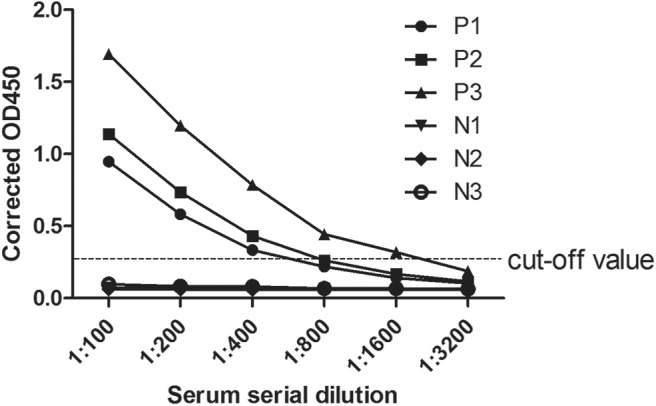
Anti-CanineCV antibody titers in positive and negative canine serum samples. Positive and negative canine serum samples were prepared in dilutions of 1:100, 1:200, 1:400, 1:800, 1:1,600, and 1:3,200 used in an ELISA assay. The cutoff was determined by counting the mean OD value of the ELISA negative samples plus 3 standard deviation (SD).

According to the iELISA procedure, the antibody titers were also determined by double dilution method. Three positive and negative samples were selected as examples. In Figure 4, the antibody titers of the three positive samples were 1:400, 1:800, and 1:1,600. Moreover, in positive samples the measured OD450 values exhibited an antibody concentration-dependent manner.

### Detection of CanineCV in the Serum Samples

Among the 32 ELISA-positive serum samples, 14 serum samples (34.75%) tested positive for the CanineCV DNA by qPCR, while two serum samples (5.26%) tested positive for CanineCV DNA in the 38 ELISA-negative serum samples. The calculated percentage of CanineCV DNA positive rate in the ELISA-negative serum samples was near that reported in other report, and the reported serum prevalence of the CanineCV was 3.3% (6). A statistical difference was observed between the ELISA-positive and -negative serum samples (*p* < 0.0001).

### Characteristics of Antibodies and Virus Shedding of in Experimental Infection

**Figure 5:**
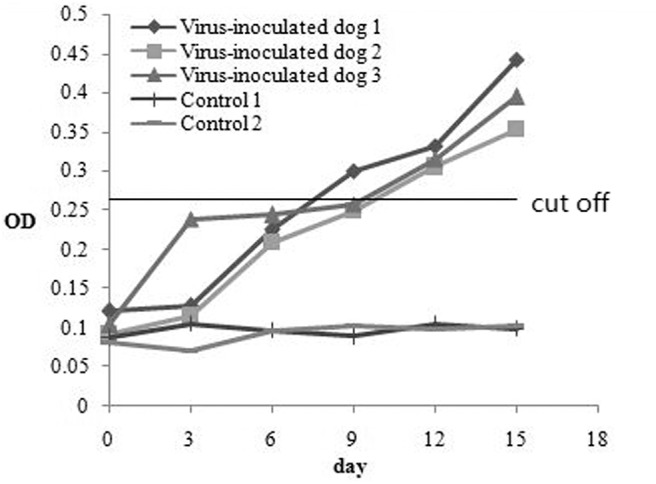
The antibody production of CanineCV in artificially experimental infected cases.

To investigate the antibody production of CanineCV in artificially infected cases, we performed experimental infection by gavage. On the first 3 days, the puppies showed no obvious abnormality, after fourth day, the puppies showed depression. The two puppies began to develop anorexia, vomiting, and mild diarrhea, and the third puppy developed diarrhea on the 10th day. The two puppies died on the 15th day. The results of viral detection are shown in Table 6. From the sixth day, CanineCV was found in the fecal swabs of one puppy. After eighth days, two dogs were detected positive. From 10th day to 15th day, three puppies were detected to be CanineCV-positive. Except for that on the tenth day, no nasal swabs were detected positive for the virus. No virus was detected in oral swabs during the entire observation period. The tested antibody results by iELISA showed that one of the three dogs developed seroconversion on day ninth, and the three dogs showed positive reaction on the twelfth day (Figure 5). Dogs in the control group had no abnormalities, and no viruses or antibodies were detected.

**Table 6:** Detection of the CanineCV DNA by qPCR in the experimental infection.

**Group**	**Swabs**	**The qPCR positive number of the samples(copies/reaction)**								
		**0 d**	**2 d**	**4d**	**6 d**	**8 d**	**10 d**	**12 d**	**14 d**	**15 d**
Infection	Oral	0	0	0	0	0	0	0	0	0
	Nasal	0	0	0	0	0	1([2.75 ± 0.92] ×10^6^)^a^	0	0	0
	Fecal	0	0	0	1 ([5.75 ± 1.84] ×10^6^)^a^	2 ([3.62 ± 1.45] ×10^6^)^a^ ([1.70 ± 1.72] ×10^6^)^b^	3 ([4.27 ± 1.68] ×10^7^)^a^ ([2.75 ± 1.47] ×10^7^)^b^ ([3.63 ± 1.86] ×10^7^)^c^	3 ([5.42 ± 1.62] ×10^8^)^a^ ([3.09 ± 1.49] ×10^8^)^b^ ([1.64 ± 1.91] ×10^8^)^c^	3 ([7.26 ± 1.98] ×10^7^)^a^ ([9.12 ± 2.39] ×10^7^)^b^ ([10.23 ± 1.75] ×10^7^)^c^	3 ([3.41 ± 2.85] ×10^7^)^a^ ([2.24 ± 1.98] ×10^7^)^b^ ([4.58 ± 2.43] ×10^7^)^c^
Control	Oral	0	0	0	0	0	0	0	0	0
	Nasal	0	0	0	0	0	0	0	0	0
	Fecal	0	0	0	0	0	0	0	0	0

a *The detection result of the No.1 dog in the infected group*.

b *The detection result of the No.2 dog in the infected group*.

c *The detection result of the No.3 dog in the infected group*.

## Discussion

Serological assays to measure virus specific IgG or IgM are important to identify infection, serological epidemiology is an important technique used to understand the infection (17, 18). The increasing incidence of CanineCV infection may become an actual threat to the canine health. CanineCV has been reported in the US (5, 6), Germany (GenBank accession number: KF887949), Italy (4), and China (7, 9). At present, no rapid and reliable serodiagnostic test has been developed to detect antibody for CanineCV infection because of difficulty in culturing the virus. No cell culture system has successfully determined CanineCV.

The capsid protein of circovirus is a useful antigen for serodiagnosis in infected animals with relatively high specificity and sensitivity (16, 19–23). As the only circovirus structural protein and the major immunogenic protein (24, 25), Cap is a major viral target or useful tool for epidemiological surveys (14, 23, 26, 27). To our knowledge, this study is the first to use iELISA on recombinant capsid proteins for diagnosis of CanineCV infection. Considering the facts that PCV2 can infect canine (28), we deleted the first 40 amino acid residues at the amino terminal to avoid the possible cross-reaction of the two viruses in future detection. The amino acid sequences of PCV2 Cap, especially in the first 40 amino acid residues at the amino terminal, are highly homologous to those of CanineCV. According to the previous reports, the first 40 amino acid residues at the amino terminal is unlikely to affect protein antigenicity (14, 29).

In this study, the truncated *cap* gene was cloned and expressed in *E. coli* because bacterial expression systems are more convenient. By using the purified rCap as a diagnostic antigen, we developed a specific and sensitive iELISA to identify CanineCV infection. The optimal working conditions were also determined by utilizing the checkerboard ELISAs. Our data also demonstrated the high specificity of this antigen, which did not react with antibodies against CAV2, *CPV2*, RV, *CDV, E. coli*, and *S. typhimurium*. The intra- and inter-assay CVs were satisfactorily low.

No standard methods for CanineCV antibody detection have been developed. Hence, we used Western blot as a reference test to validate the sensitivity and specificity of the rCap iELISA for detection of CanineCV*-*specific antibodies in dogs. No significant difference in the results was observed between ELISA and Western blot (*p* > 0.05). A kappa of 0.796 was statistically obtained between iELISA and Western blot, resulting in a good agreement between these assays (30). In Comparison with Western blot for large number of samples, ELISA is time saving, convenient, and fast and can be used to quantify precise antibody levels. Thus, the iELISA with high levels of sensitivity, specificity, and reproducibility was developed successfully for detection of antibodies to CanineCV. This assay is applicable for screening large numbers of serum samples.

To further confirm the potential application of the rCap ELISA in CanineCV antibody detection and epidemiological investigation, we performed a serological survey using the assay on samples from five cities in northeastern China. The total positive rate of CanineCV antibodies was 39.82% in the samples. Some differences in the prevalence of the CanineCV infection were observed among different cities, but the difference was not significant (*p* > 0.05). Obvious differences in the positive rate were observed in different months in the same area. The values in October, November, March, April were significantly higher than those in May, June, December, January. Overall, CanineCV infection has high incidence and is widespread in northeastern China. Furthermore, the correlation of diarrhea history in the past half of year with a seropositive test for CanineCV was calculated and showed a significant difference in the samples with or without diarrhea history. This result is consistent with the conclusion of Hsu et al. (9), who reported that the difference in CanineCV prevalence was highly significant (*p* < 0.001) between diarrheal and health dogs, as detected by qPCR.

Analysis of the correlation of age with seropositive rate of CanineCV showed the higher prevalence of CanineCV infection in older dogs. By contrast, CanineCV infection was more frequent in younger dogs in diarrheal group. The possible reason is that the target substances differ, that is, we detect CanineCV antibodies and they detect DNA, and it is presumed that the older dogs are more likely to be infected, and the antibodies can exist for a long time in the dogs. From another point of view, the two studies confirmed a significant difference between healthy animals and dog suffering from diarrhea. The virus plays a role in diarrhea. A recent work, on the pathogenetic role of CanineCV in dogs with gastroenteritis showed that it had a limited role in the development of acute gastroenteritis (31). The pathogenicity should be explored further.

Few factors might have caused bias to our analysis. First, no authoritative assay or third assay is not available to carry out a confirmatory test. We had to use the methods established in this study to validate the sensitivity, specificity, and accuracy of the iELISA. A good consistency was found between iELISA and Western blot. Furthermore, all the samples used for statistical analysis were collected from the Animal Teaching Hospital, which provided detailed and accurate records. However, the sample number was limited, especially in healthy animal samples. In addition, most of the clinical samples were obtained from animal clinics. Hence, most of the dogs were sick or dead. The dogs differed in terms of physiological or pathological conditions, and the course of the disease could not be determined. Lastly, the serum samples obtained from a clinic might have been obtained from the same dog for multiple visits. Hence, samples were obtained from the same dog at different times, resulting in similar results.

Based on the results of previous studies and the results of this study, we seem to draw the following deduces: (1) CanineCV may be a common virus in dogs and is opportunistic, and can cause secondary infection only, or even does not cause disease in dogs; (2) CanineCV has two different types: pathogenic and non-pathogenic; (3) CanineCV, as a co-infection factor, is associated with diarrhea only in the presence of other pathogens such as parvovirus or other viruses.

Hence, CanineCV infections are probably endemic in dogs from northeast provinces in China. However, the seroprevalence of dogs in other areas in China and feeding environments, such as farm and wild, should be detected. Next, the regular pattern of antibody production curve should be detected in a single artificial or natural infection case. Moreover, the pathogenicity of CanineCV infection, the relationship between antibody level and immune protection, and the harmful effects of this virus should be further investigated.

## Conclusions

ELISA based on affinity-purified rCap protein of CanineCV was useful for detection of antibodies to this circovirus in infected dogs with high levels of specificity and sensitivity. This method showed potential for serological diagnosis, large-scale serological surveys, and antibody titer monitoring of CanineCV infection. This report is the first to demonstrate that CanineCV infection is common in the dog population in northeastern China. The results showed obvious differences in the positive rate associated with diarrhea, age and season, but not with different cities. Our findings also provide basis for evaluating the pathogenic potential of CanineCV. However, the pathogenicity, the relationship between antibody level and immune protection, and the harmful effects of this virus remain to be established.

## Data Availability Statement

The data analyzed during the current study are available from the corresponding author on reasonable request.

## Ethics Statement

This study was carried out in accordance with the recommendations in the Guide for the Care and Use of Laboratory Animals of the Ministry of Health, China, and approved by Harbin Veterinary Research Institute (Approved No. Heilongjiang-SYXK-2006-032). Samples were collected only from animals for laboratory analyses, avoiding unnecessary pain and suffering of the animals. The owners gave their written consent for sample collection, and the locations where we sampled are not privately owned or protected in any way. The studies did not involve endangered or protected species.

## Author Contributions

JG and HC: conceived and designed the experiments. YS, JG, ZW, XC, and LZ: performed the experiments. JG, YS, and LZ: analyzed the data. LZ, HL, YW, SW, and WC: contributed reagents, materials, and analysis tools. JG and ZW: wrote the paper. All authors read and approved the final manuscript.

## Conflict of Interest

The authors declare that the research was conducted in the absence of any commercial or financial relationships that could be construed as a potential conflict of interest.
